# Acquired Bilateral Seminal Vesicle Cysts: A Rare Manifestation of Prostate Cancer

**DOI:** 10.7759/cureus.5842

**Published:** 2019-10-05

**Authors:** Nassib Abou Heidar, Moutafa Moussally

**Affiliations:** 1 Urology, American University of Beirut Medical Center, Beirut, LBN; 2 Surgery, American University of Beirut Medical Center, Beirut, LBN

**Keywords:** seminal vesicle cysts, multiple seminal vesicle cysts, prostate cancer

## Abstract

Seminal vesicle cysts are rare clinical entities estimated to occur in 0.005% of the male population. These cysts could be either congenital or acquired, primary or secondary. The majority of these cysts are asymptomatic and discovered incidentally. Larger cysts could present with a wide array of symptoms such as dysuria, acute urinary retention, perineal discomfort, or hematospermia. We hereby present a case of bilateral seminal vesicle cysts arising due to prostate adenocarcinoma. We discuss the presentation of such cysts, their method of diagnosis, and the approach to management.

## Introduction

Seminal vesicle cysts are uncommon entities estimated to occur in 0.005% of the male population [1]. The cysts could be either congenital or acquired. Acquired cysts of seminal vesicles could be due to iatrogenic obstruction of the ejaculatory duct or secondary to prostatitis or malignancy [2]. The majority of seminal vesicle cysts are asymptomatic and are discovered incidentally while larger cysts tend to be symptomatic [3]. The symptoms caused by such cysts are related to obstruction and local compression, including dysuria, urinary frequency, urgency, hematospermia, painful ejaculation, perineal discomfort, or suprapubic pain [3-4]. We hereby present a case of bilateral seminal vesicle cysts secondary to prostate cancer.

## Case presentation

**Figure 1:**
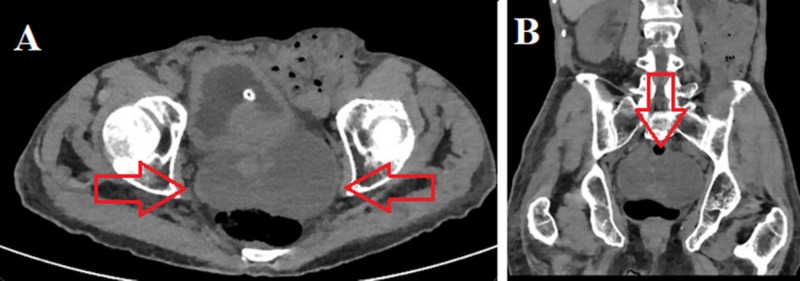
Non-contrast CT scan images, axial and coronal cuts Non-contrast CT scan images showing bilateral, large 8-cm seminal vesicle cysts (red arrows)

**Figure 2:**
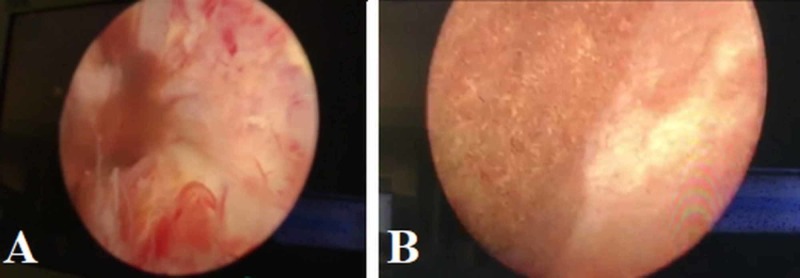
Cystoscopic view Neoplastic outgrowth at the location of the verumontanum (A); after resection of the prostatic neoplasm, the cystoscope was entered into the seminal vesicle with old blood and seminal fluid identified (B)

An 84-year-old gentleman with a medical history pertinent for controlled hypertension presented with a one-month history of urinary retention requiring catheterization refractory to tamsulosin and failing voiding trials multiple times. His history of the present illness was also pertinent to an 8-kg weight loss in the past two months and decreased oral intake, as well as anejaculation for more than a year. On a general physical exam, he appeared cachectic but was alert and oriented. His abdominal exam revealed a soft, non-tender abdomen and rectal examination revealed a very hard and large non-tender prostate. Workup was initiated with a serum creatinine of 1.5 mg/dL and a prostate-specific antigen of 86 ng/mL. A computed tomography (CT) scan revealed a very large prostate with bilateral fluid collections measuring 8 cm x 8 cm x 7 cm in the largest dimensions at the area of the seminal vesicles (Figure 1). Next, it was decided to perform a transurethral resection of the prostate for tissue diagnosis and to relieve his urinary obstruction. During the transurethral resection, the prostatic tissue was noted to be sponge-like with more outgrowing tissue in the area just proximal to the verumontanum (Figure 2). After performing resection of this tissue, dark fluid started draining and after completion of resection, the cystoscope was inside the seminal vesicle cysts mentioned in the CT scan containing old blood mixed with seminal fluid; this was performed at both sides. The patient tolerated the procedure well and had a urethral catheter for two days, after which the patient successfully voided after removal. The pathologic diagnosis of the tissue was Gleason 8(4+4) prostate adenocarcinoma with a cribriform pattern. The patient was started on androgen deprivation in preparation for external beam radiation to the prostate.

## Discussion

Acquired cysts within seminal vesicles are rare occurrences; however, with more prevalent and advanced imaging, their detection is improving. They are most prevalent in men between the ages of 20 and 40 years [5]. The majority of cases of seminal vesicle cysts are congenital and are usually associated with genitourinary malformations due to the common embryonic origin. These urinary deformities include hypospadias, cryptorchidism, hermaphroditism, or unilateral renal agenesis. When congenital seminal vesicle cysts are associated with ipsilateral renal agenesis, it is referred to as Zinner syndrome [2,4]. Congenital cysts usually arise in utero between the fourth and seventh weeks of gestation from the primitive mesonephric duct [1]. On the other hand, acquired seminal vesicle cysts stem from the obstruction of the ejaculatory ducts due to posterior urethral inflammation, bladder neck lesions, ejaculatory duct stones, recurrent urinary tract infections, or stricture secondary to prostate surgery [2,4-5].

The majority of seminal vesicle cysts tend to be asymptomatic especially those with a size below 5 cm. However, larger cysts whose size exceeds 8 cm are likely to cause symptoms due to mass effect and can be palpated on a physical exam [3]. These cysts are usually diagnosed around the age of sexual activity due to the accumulation of seminal fluid in the vesicles secondary to incomplete drainage [3]. Symptoms include urinary frequency, dysuria, perineal discomfort, hematospermia, painful ejaculation, acute urinary retention, and recurrent epididymitis [3-4]. In a series of seven patients reported by Wang et al., perineal and lower abdominal discomfort was the most common symptom, occurring in six patients [4]. By compressing adjacent viscera and structures, seminal vesicle cysts can result in a urinary obstruction similar to our patient. In severe cases, this can lead to obstructive nephropathy and subsequent hydronephrosis [1]. Moreover, a case of a lethal infected seminal vesicle was reported in the literature [6].

As such, the diagnosis of seminal vesicle cysts poses a considerable challenge due to the vague presenting symptoms and the possible resulting complications. The presence of seminal vesicle cysts should be suspected in males with unexplained urinary frequency or hematospermia [5]. Furaya et al. reported that seminal vesicles were the origin of 52% of hematospermia in their study [7]. In addition, the study showed that 82% of patients with hematospermia had seminal vesicle abnormalities [5,7]. As such, the presence of hematospermia warrants considering seminal vesicle cysts as part of the differential.

Imaging modalities, such as computed tomography (CT), magnetic resonance imaging (MRI), transrectal ultrasonography (TRUS), cystoscopy, vasovesiculography, and intravenous urography, can all be used to diagnose seminal vesicle cysts [3-4]. MRI comprises the optimal imaging modality to identify seminal vesicle cysts. Although both MRI and CT allow the accurate description and visualization of pelvic cysts in relation to the seminal vesicles, MRI has better diagnostic accuracy than CT scans [4]. Seminal vesicle cysts appear as thin-walled cysts with regular margins on CT [4-5]. In addition, a study showed that MRI was superior to TRUS in the diagnosis of seminal vesicle and prostate abnormalities [8].

The approach to the management of seminal vesicle cysts is dependent on their size and symptomatology. Small or asymptomatic cysts can be managed conservatively. However, surgical intervention is warranted for large cysts with compression effects. Transurethral resection with unroofing of the cyst is the treatment of choice due to the low complication rate and simplicity of the procedure [3-4]. In a series of seven patients, Wang et al. were able to demonstrate the safety of transurethral unroofing. None of the patients on whom the procedure was done developed any complications [4]. Transrectal aspiration is another possible intervention that might relieve symptoms [6]. The difficulty of the open surgical approach and the location of the seminal vesicles deep in the pelvis render open surgery an unfavorable option [4]. Laparoscopic or robotic-assisted resection of seminal vesicle cysts has been described in the literature as a valid option for the treatment of non-malignant seminal vesicle conditions without any morbidity [9]. Laparoscopic resection is associated with a shorter hospital stay, less pain, and quicker recovery. However, it is associated with infertility and ureteral damage due to the proximity of the seminal vesicles to the ureter and neurovascular bundle.

Our case is one of the few in the literature that discusses the formation of bilateral, acquired large seminal vesicle cysts due to prostate adenocarcinoma. The treatment modality chosen was best in our minds since it treated the urinary obstruction as well as drained the seminal vesicles. Furthermore, pathologic confirmation of high-grade prostatic adenocarcinoma was possible after tissue examination, allowing the initiation of androgen deprivation therapy. However, asymptomatic patients are treated conservatively due to the benign nature of seminal vesical cysts.

## Conclusions

Seminal vesicle cysts are rare entities that are often overlooked or missed. While the majority of seminal vesicle cysts are asymptomatic and discovered incidentally, larger cysts can result in dysuria, urinary retention, perineal discomfort, or hematospermia. The diagnosis of this condition remains a challenge. CT or MRI scans are the diagnostic tests of choice. Treatment modalities are dependent on the size of the cyst, as well as symptoms and the full clinical picture of the patient. Asymptomatic patients are treated conservatively. Whilst laparoscopic excision of seminal vesicle cysts has been fairly described and is safe, the transurethral unroofing of cysts remains the optimal treatment option.
